# Prognostic effect of CD74 and development of a radiomic model for predicting CD74 expression in non-small cell lung cancer

**DOI:** 10.3389/fmed.2025.1586253

**Published:** 2025-05-21

**Authors:** Yancheng Wang, Zhen Gao, Meng Li, Zhen Feng, Hui Wang

**Affiliations:** ^1^Department of Thoracic Surgery, Shandong Provincial Hospital Affiliated to Shandong First Medical University, Shandong First Medical University, Jinan, China; ^2^Department of Thoracic Surgery, Shandong Provincial Hospital, Shandong University, Jinan, China

**Keywords:** CD74, non-small cell lung cancer, radiomics, machine learning, prediction model

## Abstract

**Background:**

The classical prognostic indicators of lung cancer are no longer sufficient for prognostic stratification and individualized treatment of highly heterogeneous non-small cell lung cancer (NSCLC). This study aimed to establish a radiomics model to predict CD74 expression level in NSCLC patients and to explore its role in the tumor immune response and its prognostic value.

**Methods:**

The prediction model was developed based on 122 NSCLC transcriptome samples, including 68 paired enhanced CT and transcriptome samples. Survival analysis, gene set variation analysis, and immune cell infiltration analysis were used to investigate the relationship between CD74 expression and tumor immune response. Logistic regression (LG) and support vector machine (SVM) analysis were used to construct the prediction model. The performance of the model was assessed with respect to its calibration, discrimination, and clinical usefulness.

**Results:**

High CD74 expression is an independent prognostic factor for NSCLC and is positively correlated with antigen presentation and processing gene expression and antitumor immune cell infiltration. The radiomics prediction models for CD74 expression demonstrated good predictive performance. The areas under the receiver operating characteristic curves for the LG and SVM radiomics models were 0.778 and 0.729, respectively, in the training set and 0.772 and 0.701, respectively, in the validation set. The calibration and decision curve analysis curves demonstrated good fit and clinical benefit.

**Conclusion:**

CD74 expression significantly impacts the prognosis of NSCLC patients. The radiomics model based on contrast-enhanced CT exhibits good performance and clinical practicability in predicting CD74 expression.

## Introduction

Lung cancer ranks first among cancers in terms of mortality and second in terms of overall morbidity (1). Among the various types of lung cancer, non-small cell lung cancer (NSCLC) is the most common (2, 3). Surgical resection is considered the gold standard treatment for early-stage lung cancer; however, the surgical outcome and prognosis for advanced patients are poor (4). Therefore, for advanced lung cancer patients who are not eligible for surgery, it is essential to predict their prognosis and provide precision treatment for long-term high-quality survival with tumors (5). NSCLC exhibits significant genetic and cellular heterogeneity (6). There is an urgent need for individualized therapy in NSCLC. Traditional diagnostic and prognostic indicators for lung cancer include clinicopathological features, laboratory diagnostic indicators such as carcinoembryonic antigen and carbohydrate antigen 125, and CT imaging methods (7, 8). However, these indicators are no longer sufficient to meet the clinical requirements of precision medicine (2, 9). Therefore, it is necessary to further explore new prognostic indicators to meet the precision treatment needs of NSCLC.

Analysis of gene expression can identify novel markers and targets for patient management and treatment. Several studies have suggested that CD74 may serve as a prognostic factor (10, 11) and therapeutic target (12, 13) for patients with malignant tumors. CD74 encodes class II major histocompatibility complex-associated proteins (13, 14), which are primarily involved in antigen presentation during the immune response (15). Additionally, CD74 can act as a cell surface receptor for macrophage cytokines, mediating the survival and proliferation of macrophages (14). For example, one study demonstrated that CD74 is essential for the distant metastasis of breast cancer, and targeting CD74 therapy may be an effective strategy for breast cancer treatment (16). Furthermore, CD74-ROS1 is the most common form of ROS1 fusion in NSCLC, and CD74-NRG1 gene fusion activates genomic alterations in aggressive mucinous adenocarcinomas, offering potential therapeutic opportunities for lung tumor subtypes that have not yet been effectively treated (17). Currently, the expression level of CD74 can only be detected through peripheral blood cytokine analysis, mRNA or protein level analysis using fresh tissue samples, or paraffin tissue sample analysis, but these methods are expensive and complex, have limited reflection of tumor parenchyma, and are prone to bias.

RNA-seq offers high resolution and low technical variability (18), and demonstrates a high degree of concordance with other gold-standard techniques in transcriptomics, both for absolute and relative gene expression measurements (19). However, its high cost and the invasive nature of sample collection limit its clinical applicability. Immunohistochemistry (IHC), by contrast, is more affordable but suffers from operator variability and antibody bias, leading to inter-laboratory heterogeneity and the lack of quantitative, objective assessments (20). Given these limitations, imaging techniques provide distinct advantages. Previous studies have shown that radiomics can be used to noninvasively predict the pathological type or molecular features of NSCLC by extracting high-throughput features from images for quantitative analysis (21–23). Additionally, it also can effectively identify patients at high risk of disease recurrence and positively improve NSCLC stratification and patient survival through noninvasive prediction of gene expression (18–20). Based on these studies, radiomics may be a powerful tool for facilitating decision making in the individualized management of NSCLC.

In view of the advantages of radiomics, this study used radiomics techniques to predict CD74 mRNA expression in NSCLC tumor tissues and combined them with bioinformatics analysis to explore the molecular mechanism of the tumor immune response related to CD74 expression. This approach provides a convenient and noninvasive new indicator for the stratification and optimal individualized treatment of NSCLC patients.

## Patients and methods

### Patients

**Figure 1:**
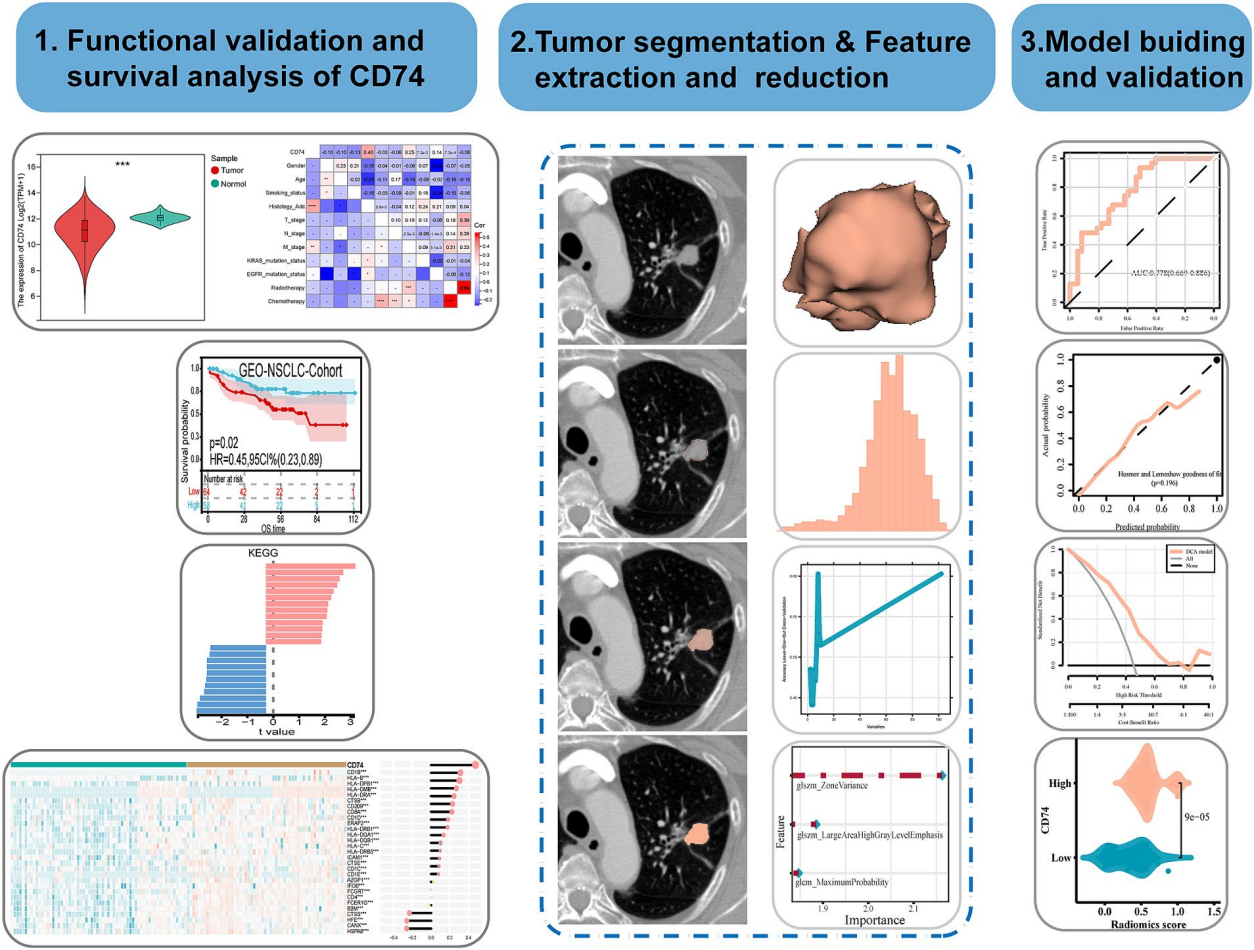
Flow chart of this study.

The flow chart of this study is shown in Figure 1. This workflow is shown in the Graphical Abstract. The NSCLC cohort of this study included medical imaging data from the NSCLC Radiogenomics dataset in The Cancer Imaging Archive (TCIA) Public Access-Cancer Imaging Archive Wiki. The RNA-seq data and clinical follow-up data for the main cohort are from the Gene Expression Omnibus (GEO) database, and the dataset is named GSE103584 (24).^1^ The inclusion and exclusion criteria are detailed in Supplementary Table S1. Finally, 122 transcriptome samples with complete clinical information and 68 imaging samples with complete clinical information and transcriptome information were obtained. Lung adenocarcinoma (LUAD) cohort transcriptome data were obtained from the TCGA database.^2^ The inclusion and exclusion criteria are detailed in Supplementary Table S2. Finally, 320 transcriptome samples were obtained. All transcriptome data were converted to TPM format, and then log2 conversion was performed.

To determine the optimal cut-off value for CD74 expression, we used the surv_cutpoint function from the R package “survminer,” applying the maximally selected rank statistics (also known as the minimum *p*-value method) to automatically identify the expression threshold that most significantly distinguishes survival differences. This method has been used in several high-quality studies because of its sensitivity to survival differences and well-balanced grouping (25–27). This cut-off value was then used to classify patients into high and low CD74 expression groups. Consequently, the cut-off value for CD74 expression in the NSCLC cohort was determined to be 8.7430, and patients were divided into a high expression group and a low expression group accordingly. The clinical baseline characteristics of the NSCLC cohorts are detailed in Table 1. The cut-off value for the CD74 expression level in the LUAD cohort was 9.5861, and the patients were divided into a high expression group and a low expression group. The clinical baseline characteristics are shown in Supplementary Table S3. The cut-off value of the CD74 expression level in the radiomic cohort was 8.7430, and the samples were divided into a high expression group and a low expression group.

**Table 1:** Demographic and clinicopathological characteristics of patients (NSCLC).

Variables	Total (*n* = 122)	Low (*n* = 64)	High (*n* = 58)	*p*
Gender, *n* (%)				0.741
Female	33 (27)	16 (25)	17 (29)	
Male	89 (73)	48 (75)	41 (71)	
Age, *n* (%)				0.176
≤65	44 (36)	19 (30)	25 (43)	
>65	78 (64)	45 (70)	33 (57)	
Smoking_status, *n* (%)				0.103
Nonsmoker	20 (16)	11 (17)	9 (16)	
Current	24 (20)	17 (27)	7 (12)	
Former	78 (64)	36 (56)	42 (72)	
Histology, *n* (%)				<0.001
Adenocarcinoma	92 (75)	39 (61)	53 (91)	
Squamous cell carcinoma	30 (25)	25 (39)	5 (9)	
T_stage, *n* (%)				0.75
T2/T3/T4	66 (54)	36 (56)	30 (52)	
Tis/T1	56 (46)	28 (44)	28 (48)	
N_stage, *n* (%)				0.863
N0	97 (80)	50 (78)	47 (81)	
N1/N2	25 (20)	14 (22)	11 (19)	
M_stage, *n* (%)				0.022
M0	117 (96)	64 (100)	53 (91)	
M1	5 (4)	0 (0)	5 (9)	
KRAS_mutation_status, *n* (%)				0.145
Mutant	23 (19)	9 (14)	14 (24)	
Unknown	27 (22)	18 (28)	9 (16)	
Wildtype	72 (59)	37 (58)	35 (60)	
EGFR_mutation_status, *n* (%)				0.071
Mutant	18 (15)	8 (12)	10 (17)	
Unknown	28 (23)	20 (31)	8 (14)	
Wildtype	76 (62)	36 (56)	40 (69)	
Radiotherapy, *n* (%)				1
No	108 (89)	57 (89)	51 (88)	
Yes	14 (11)	7 (11)	7 (12)	
Chemotherapy, *n* (%)				0.521
No	86 (70)	43 (67)	43 (74)	
Yes	36 (30)	21 (33)	15 (26)	

### Survival analysis

Univariate Cox regression and multivariate Cox regression survival analyses were performed for each variable. A Kaplan–Meier survival curve was used to show the difference in the survival rates in different groups, and the log-rank test was used to test the significance of differences in the survival rates among groups. Univariate Cox regression was used to analyze the effect of CD74 expression on prognosis in different subgroups of covariates. The interaction between CD74 expression and other covariates was analyzed using the likelihood ratio (OR value) test. Cox regression analysis and survival analysis were performed using the R packages “survival” and “forestplot,” and the R package “survminer” was used to summarize and visualize the results.

### Gene set variation analysis (GSVA) and correlation analysis between CD74 high and low subgroups

GSVA is mainly used to evaluate the results of gene set enrichment in the transcriptome (28). It is mainly used to transform the expression matrix of genes between different samples into the expression matrix of gene sets between samples to evaluate whether different pathways are enriched in different samples. The enrichment scores of KEGG pathway gene sets and hallmark gene sets^3^ in the NSCLC cohort and LUAD cohort samples were calculated by the GSVA algorithm. The R package “limma” was used to analyze the difference in the pathway enrichment score between the high and low CD74 groups, and the different paths were visualized, with |*t*| = 1 as the critical value.

### Immune-related analysis associated with CD74 expression

The Wilcoxon rank sum test was used to detect the differential expression of antigen processing and presenting genes between the high and low CD74 groups. Genes with *p* < 0.001 were visualized, and the results are displayed in a heatmap. The gene expression matrix of NSCLC samples and LUAD samples was uploaded to the CIBERSORTx database^4^ to calculate the immune cell infiltration of each sample (29). The R package “corrplot” was used to analyze the correlation between CD74 expression and the degree of immune cell infiltration.

### CT imaging parameters and image processing

CT imaging parameters included a slice thickness ranging from 0.625 to 3 mm (median: 1.5 mm), an X-ray tube current between 124 and 699 mA (mean 220 mA), and a tube voltage ranging from 80 to 140 kVp (mean 120 kVp) (24).

To minimize the variability caused by differences in scanning equipment, imaging protocols, and lesion sizes, a series of standardized preprocessing steps were applied in this study. All CT images were resampled using the ‘sitkBSpline’ interpolator to achieve an isotropic voxel size of 1 × 1 × 1 mm^3^, thereby reducing variability related to scanning parameters and lesion dimensions. Voxel intensity values were discretized using a fixed bin width of 25 HU to reduce image noise and standardize signal intensity, enhancing the stability of radiomic features across different images. Image normalization was performed by scaling signal intensities to a range of 1–500 HU, aiming to minimize intensity variations across images acquired from different machines and further improve data consistency. Additionally, gray-level values were standardized using Z-score normalization to adjust the gray-level distributions across images, reduce inter-patient variability, and enhance the stability of feature computation.

### Region of interest (ROI) of image construction and consistency evaluation

3D Slicer software (version 4.10.2) was utilized by an experienced radiologist with over 10 years of expertise in diagnosing chest disease imaging, as well as another radiologist with more than 5 years of experience, to manually outline the entire area of interest to obtain the complete tumor area. In cases where there was disagreement, a consensus was reached through discussion with a more senior imaging physician. The consistency of the image features extracted from the volume of interest (VOI) delineated by the two physicians was assessed using the intraclass correlation coefficient (ICC). To further validate the results, a random sample of 20 cases was chosen using the “random number table method” and assessed by an imaging doctor with more experience.

In this study, radiomics features were extracted using Pyradiomics,^5^ including 14 shape features, 18 first-order features, and 75 s-order features, resulting in a total of 107 original radiomics features. The second-order features include GLCM, GLRLM, GLSZM, NGTDM, and GLDM, which are among the most commonly used features in radiomics research. Features with an ICC value of ≥0.75 were selected for the subsequent feature screening process (30–32).

### Radiomic feature screening

Prior to model construction, we initially applied the Recursive feature elimination (RFE) method to perform a preliminary screening of the predictors by ranking radiomic features with an ICC ≥ 0.75. RFE iteratively trains the model and eliminates features of lower importance after each iteration until the optimal subset of features is identified (33). Based on the preliminary screening, stepwise regression combined with the Akaike information criterion (AIC) was subsequently employed for secondary feature selection (34). Using AIC to balance model complexity and goodness of fit, a bidirectional stepwise regression approach was applied to further eliminate features that contributed little to the model or showed high multicollinearity. Ultimately, three representative and stable radiomic features were selected for model construction, demonstrating good predictive performance and generalizability in both the training and validation cohorts.

### Construction and evaluation of the logistic regression (LR) model and support vector machine (SVM) model

The final radiomic features were fitted using the logistic regression algorithm to establish a binary prediction model for predicting CD74 expression. The logistic regression fitting was performed using the “glm” function from the R package “stats.” The radiomics model formula was calculated as the product of the feature and its corresponding coefficient plus the intercept value. Furthermore, the final screening radiomic features were fitted using the SVM algorithm to establish a binary prediction model for predicting CD74 expression. SVM algorithm fitting was performed using the R package “caret.”

To evaluate the predictive performance of the LR model and SVM model, we used the receiver operating characteristic (ROC) curve. Additionally, we performed 5-fold internal cross-validation. The fit degree of the prediction model was evaluated using the calibration curve. Moreover, we drew a decision curve analysis (DCA) to assess the clinical benefit of the prediction model.

The LR radiomic model and SVM radiomic model provided the radiomics score for each sample. We employed the Wilcoxon test to assess whether there were differences between the high and low CD74 groups in terms of radiomics score.

### Statistical analysis

The statistical analysis for this study was conducted using R 4.1.0. The t test was used for quantitative data that followed a normal distribution, while the Wilcoxon test was utilized for nonnormally distributed data. For the analysis of more than two groups, the Kruskal-Wallis test was employed as a nonparametric test, and ANOVA was used for parametric tests. The “survival” R package was used to analyze the prognostic differences between the two groups, and the significance of the prognostic differences among different groups of samples was assessed using the log-rank test. The pROC package was utilized to generate ROC curves, calculate the area under the curve (AUC), and determine confidence intervals. The DeLong test was used to compare AUC values under the ROC curve. Pearson correlation analysis was used to calculate the correlation between genes, as well as between genes and clinical traits. A *p* value less than 0.05 was considered statistically significant. For multiple hypothesis testing, the false discovery rate (FDR) was calculated using the Benjamini–Hochberg method (35).

### Radiomics workflow quality assessment

To enhance the transparency and methodological rigor of this study, we systematically evaluated the quality of the radiomics workflow based on the Minimum Information for Reporting a Radiomics Study (METRICS) standard proposed by Kocak et al. (36). The total METRICS score was 87.1%. The completed METRICS checklist is provided in Supplementary material 1 to ensure the reproducibility and robustness of the study results and to facilitate the future clinical application of the model.

## Results

### Differences in expression and clinical characteristics between CD74 expression groups

**Figure 2:**
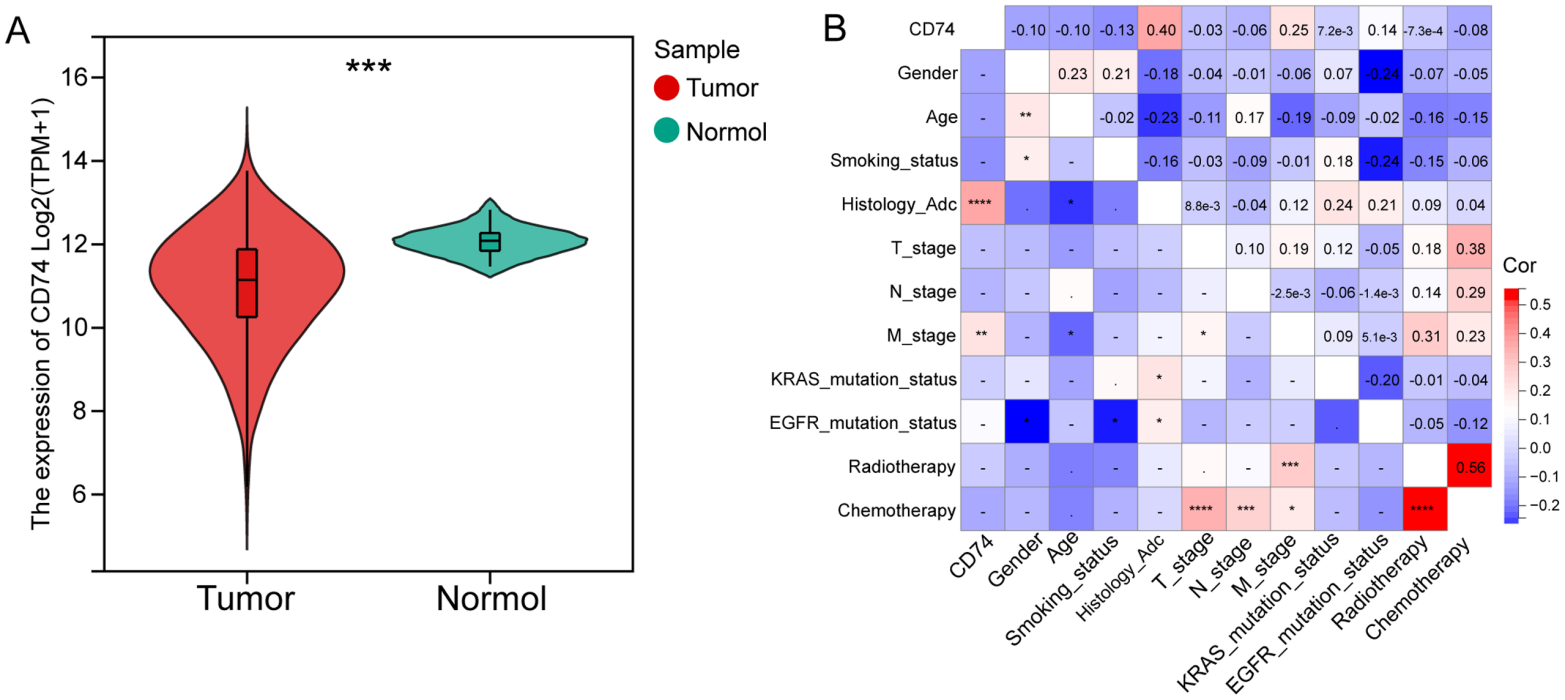
Differential expression and clinical correlation analysis of CD74. **(A)** Violin plot of differential analysis of CD74 expression in tumor and normal tissues, as shown in the figure, normal tissues showed significantly higher expression; **(B)** Heatmap of correlation between CD74 and clinical features, red represents positive correlation, blue represents negative correlation, and the higher the degree of color, the more significant the correlation. (Significant symbol: –, *p* ≥ 0.05; *, *p* < 0.05; **, *p* < 0.01; ***, *p* < 0.001; ****, *p* < 0.0001).

The expression levels of CD74 in tumor tissues and normal tissues were compared based on the RNA-seq data of lung adenocarcinoma (LUAD) and lung squamous cell carcinoma (LUSC) patients from the TCGA database. As shown in Figure 2A, the expression level of CD74 was found to be lower in tumor tissues than in normal tissues (*p* < 0.001).

The NSCLC cohort consisted of 122 patients, with 58 patients in the CD74 high expression group and 64 patients in the CD74 low expression group. The clinical information of the patients is presented in Table 1. Analysis revealed that only histological subtype showed a statistically significant difference between the high and low expression groups. There were no significant differences observed in age, sex, smoking status, T stage, N stage, M stage, EGFR mutation, KRAS mutation, chemotherapy, or radiotherapy between the two groups. Correlation analysis demonstrated a positive correlation between CD74 expression and histological subtype (*r* = 0.4, *p* < 0.0001) as well as distant metastasis (M stage) (*r* = 0.25, *p* < 0.01), as shown in Figure 2B. Further details on clinical information and clinical correlation analysis in the LUAD cohort can be found in Supplementary Table S3 and Supplementary Figures S1.

### Survival analysis between CD74 groups

**Figure 3:**
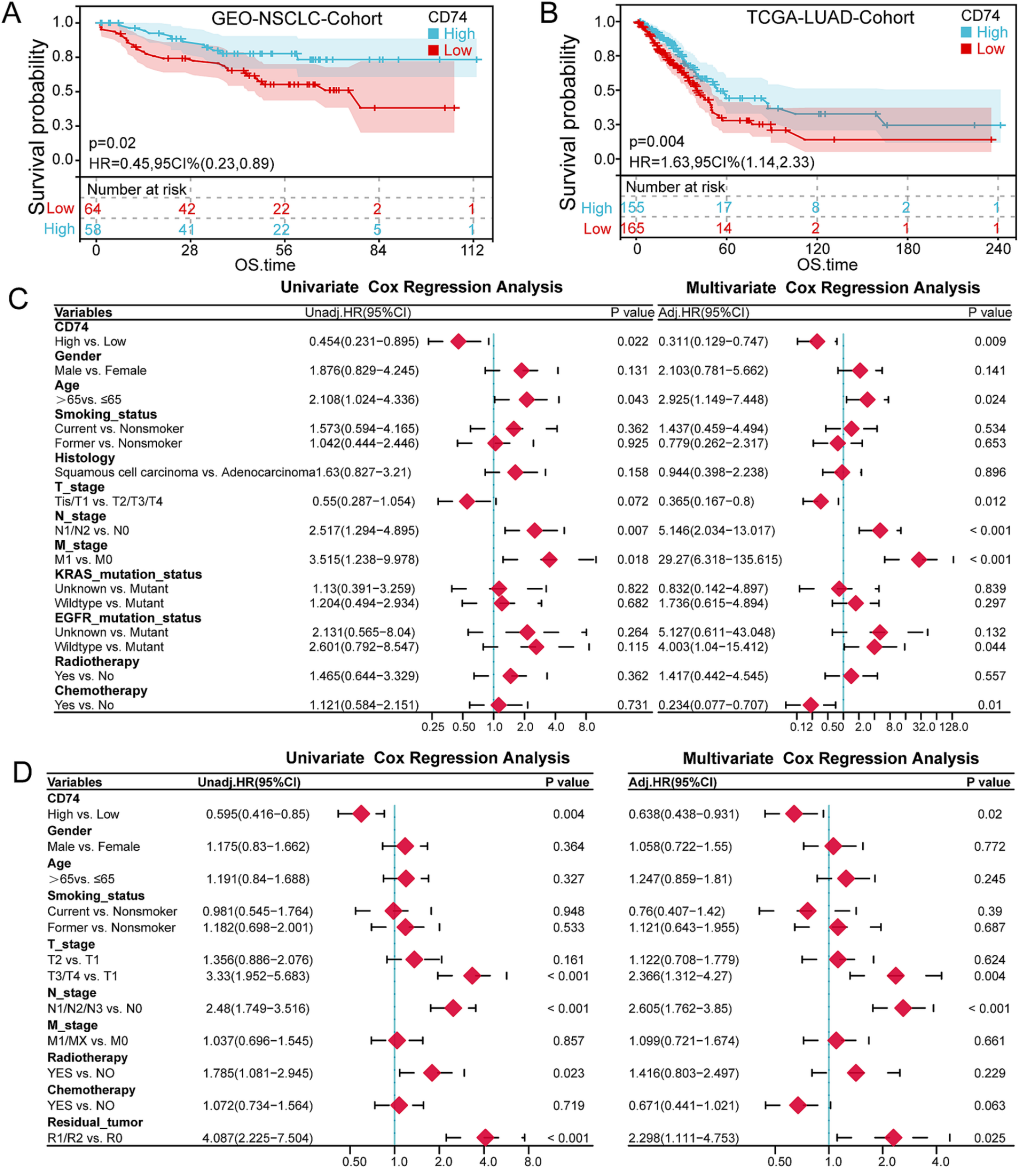
Survival analysis. **(A)** KM curve of the relationship between CD74 expression and OS in the NSCLC cohort. **(B)** KM curve of the relationship between CD74 expression and OS in the LUAD cohort. **(C)** Forest plot of univariate and multivariate COX regression analysis of CD74 and clinical characteristics in the NSCLC cohort. **(D)** Forest plot of univariate and multivariate COX regression analysis of CD74 and clinical characteristics in the LUAD cohort.

A total of 122 patients in the NSCLC cohort were included in the survival analysis. The Kaplan–Meier curve showed that high CD74 expression was associated with improved overall survival (OS) (*p* = 0.02) (Figure 3A). Similarly, in the LUAD cohort, the CD74 high expression group had a higher survival rate (*p* = 0.006) (Figure 3B). In the NSCLC cohort, patients with later N stage and M stage had worse OS (*p* < 0.005 and 0.012), and in the LUAD cohort, patients with later T stage and N stage had worse OS (*p* < 0.001) (Supplementary Figures S2a–d). Multivariate Cox regression analysis of variables showed that high CD74 expression was also a protective factor for OS (HR = 0.311, 95% CI 0.129–0.747, *p* = 0.009), which was statistically significant (Figure 3C). Similarly, in the LUAD cohort, both univariate and multivariate COX regression analyses showed that high CD74 expression was a protective factor for OS (HR = 0.595 and 0.638, 95% CI 0.416–0.85 and 0.438–0.931, respectively; *p* = 0.004 and 0.02) (Figure 3D). Therefore, high CD74 expression can be regarded as an independent prognostic factor for NSCLC and LUAD.

The interaction analysis between CD74 and other variables in the NSCLC cohort and LUAD cohort showed that the high expression of CD74 was a protective factor for OS, and there was no statistically significant difference in the interaction test of each variable (*p* > 0.05). It can be assumed that the effect of high CD74 expression on OS is the same across patients with differences in subvariables. For more details, refer to Supplementary Figure S2e.

### GSVA of CD74-related genes

**Figure 4:**
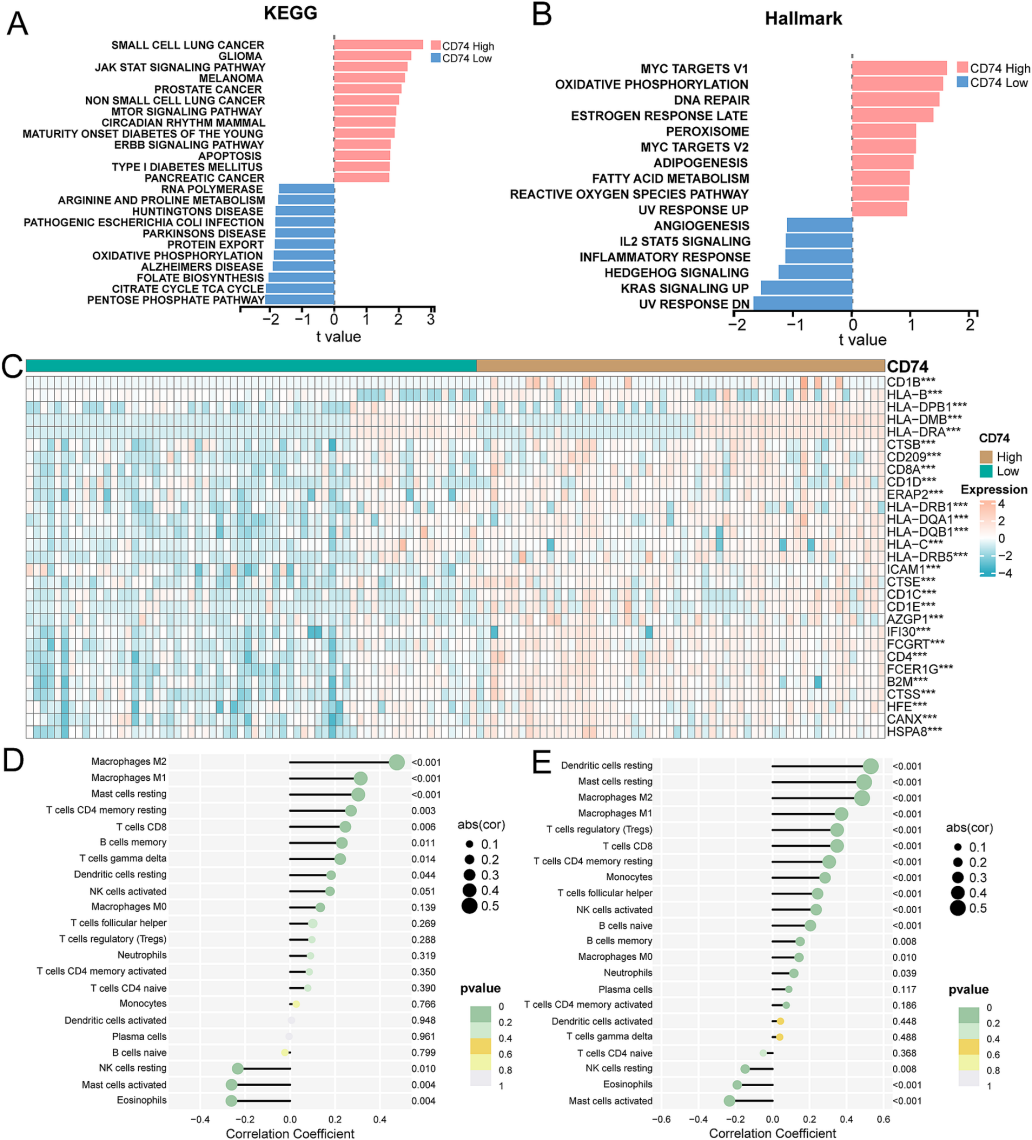
Correlation analysis of tumor pathways and immune responses for CD74 expression. **(A)** GSVA showed differential enrichment in KEGG pathway between CD74 high and low expression groups. **(B)** GSVA showed differential enrichment of Hallmarks pathways between CD74 high and low expression groups. **(C)** Heat map of differential expression of antigen processing and presentation genes between CD74 high expression group and CD74 low expression group, (significant symbol: *, *p* < 0.05; **, *p* < 0.01, ***, *p* < 0.001). **(D)** Lollipop plot of correlation between CD74 expression and immune cell infiltration in the NSCLC cohort. **(E)** Lollipop plot of correlation between CD74 expression and immune cell infiltration in the LUAD cohort.

The enrichment scores of KEGG pathway gene sets and hallmark gene sets were calculated using GSVA for the expression matrix of the NSCLC cohort and LUAD cohort. Differential analysis of the enrichment score revealed that the CD74 high expression group was significantly enriched in various cancers, such as small lung cancer, non-small cell lung cancer, pancreatic cancer, and others, within the KEGG gene set. Additionally, it was significantly enriched in signaling pathways such as apoptosis, the JAK/STAT pathway, and the ERBB signaling pathway. Please refer to Figure 4A and Supplementary Figure S3a for more details.

In the hallmark gene set, the CD74 high expression group showed significant enrichment in the DNA repair, MYC targets V1, and oxidative phosphorylation signaling pathways. Conversely, the low CD74 group exhibited significant enrichment in the Hedgehog signaling, angiogenesis, and KRAS signaling pathways. Please see Figure 4B and Supplementary Figure S3b for visual representation.

Overall, our analysis indicates that tumor cell behaviors are inhibited in the tumor microenvironment of patients with high CD74 expression, while multiple cancer pathways are activated in tumor cells with low CD74 expression.

### Analysis of antigen presentation and processing gene differences and immune cell infiltration between CD74 groups

The analysis of antigen processing and presentation gene differences between the CD74 high and low groups revealed that the gene expression levels of CD8A, CD1D, CD1C, and CD4, among others, were significantly increased in the CD74 high expression group. Please refer to Figure 4C and Supplementary Figure S3c for more details.

The expression matrices of the NSCLC cohort and LUAD cohort were uploaded to the CIBERSORTx database to calculate the level of immune cell infiltration for each sample. The correlation analysis between the level of immune cell infiltration and CD74 expression showed that CD74 was significantly positively correlated with the degree of infiltration of immune cells such as M2 macrophages, M1macrophages, resting dendritic cells, CD8 T cells, and memory B cells. Furthermore, there was a significant negative correlation between CD74 expression and resting NK cell, activated mast cell, and eosinophil infiltration (see Figures 4D,E).

### Construction of a radiomics model for predicting CD74 expression

Imaging features were extracted from 68 patients with imaging data in the NSCLC cohort. Finally, 107 radiomics features were obtained, and then the radiomics feature values were standardized. The results of the consistency evaluation showed that the median value of the ICC of radiomics features was 0.928, and there were 102 radiomics features with ICC values ≥0.75 (95.3% of all features). The features with ICC values ≥0.75 were selected by the REF method, and the top 8 features were obtained. The false positive results were removed by a stepwise regression algorithm, and finally, 3 radiomics features were obtained to construct the prediction model. The three imaging features used to construct the prediction model were glcm maximum probability, glszm large area high gray level emphasis and glszm zone variance (Table 2).

**Table 2:** Importance score of radiomics features in the model.

Features	LR-Model importance	SVM-Model importance
Glcm_Maximum Probability	1.847	0.594
Glszm_Large Area High Gray Level Emphasis	1.885	0.611
Glszm_Zone Variance	2.163	0.598

**Figure 5:**
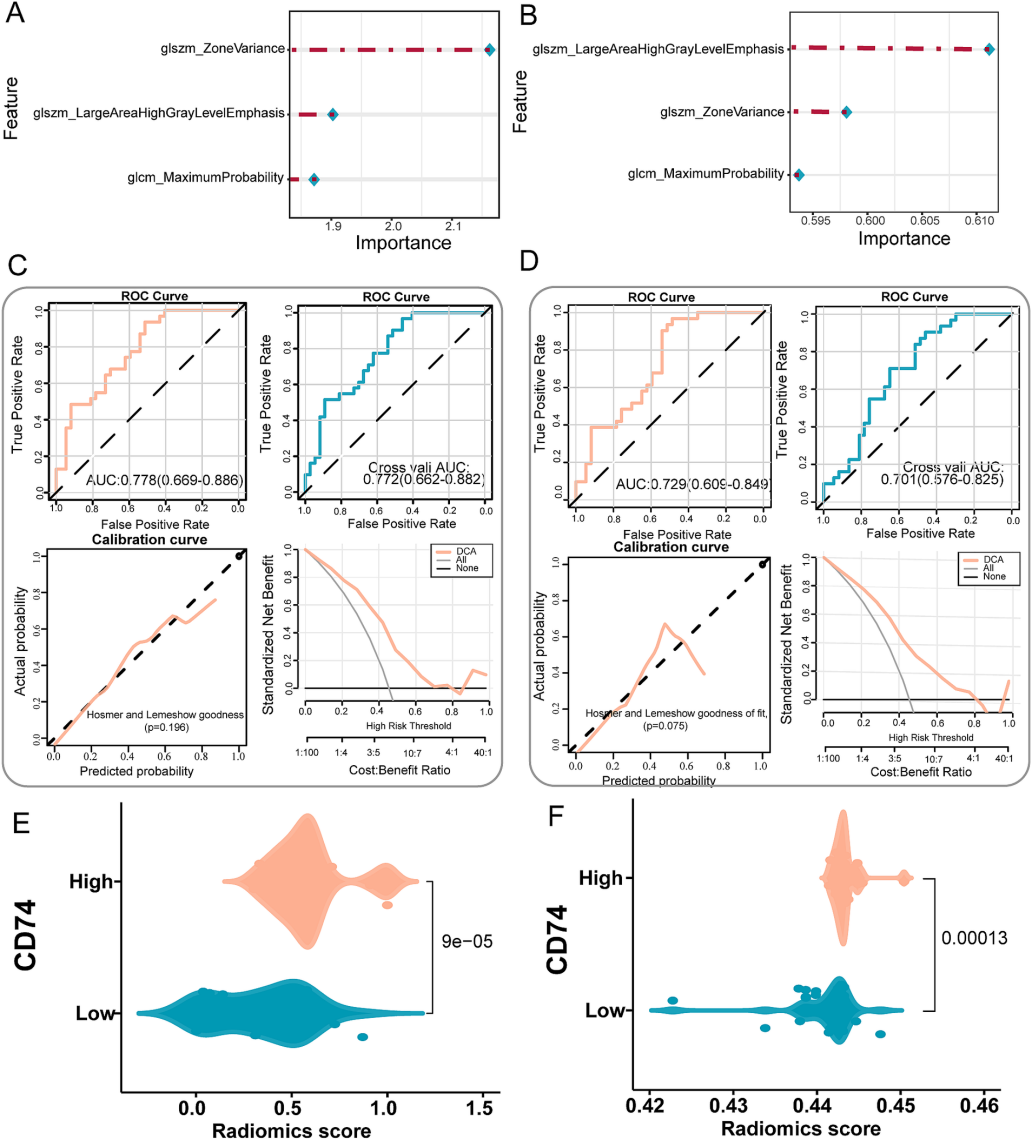
Establishment and validation of a radiomics prediction model for CD74 expression. **(A)** Importance of image features in the LR model. **(B)** The importance of image features in the SVM model. **(C)** LR model performance test, top left shows the ROC curve of model evaluation, top right shows the ROC curve of model evaluation after 5-fold cross validation, bottom left shows the Hosmer-Lemeshow goodness of fit test and calibration curve, and bottom right shows the DCA curve of model. **(D)** SVM model performance test, top left shows the ROC curve of model evaluation, top right shows the ROC curve of model evaluation after 5-fold cross validation, bottom left shows the Hosmer-Lemeshow goodness of fit test and calibration curve, and bottom right shows the DCA curve of model. **(E)** Violin plot of radiomics score differences between CD74 high and low groups in the LR model. **(F)** Violin plot of radiomics score differences between CD74 high and low groups in SVM model.

The selected radiomics features were used to construct the LR model and SVM model to predict CD74 gene expression. The importance of radiomics features in the LR model and SVM model is shown in Figures 5A,B, and the specific values are shown in Table 2. The formula of the prediction model is


$$P=1/(1+\exp(\begin{array}{c} \begin{array}{l} 33.244*\text{glszm ZoneVariance}\\ -1.414*\text{glcm MaximumProbability} \end{array}\\ \begin{array}{l} -44.301*\text{glszm Large Area High Gray}\;\\ \text{Level Emphasis} \end{array} \end{array})).$$

### Validation of the radiomics model

The performance of the LR and SVM models was evaluated using ROC curves. As shown in Figure 5C, for the LR model, the training set achieved an AUC of 0.778, with a sensitivity of 0.935 and a specificity of 0.514 at the optimal cut-off point (Table 3). In the validation set, the AUC was 0.772, with a sensitivity of 0.968 and a specificity of 0.459 (Table 3). The calibration curve and the Hosmer-Lemeshow goodness-of-fit test indicated good agreement between the predicted probabilities of high CD74 expression and the actual observations (*p* > 0.05) (Figure 5C). The decision curve analysis (DCA) demonstrated that the model had a high potential for clinical application (Figure 5C). For the SVM model, as shown in Figure 5D, the training set yielded an AUC of 0.729, with a sensitivity of 0.968 and a specificity of 0.486 at the optimal cut-off point (Table 3). In the validation set, the AUC was 0.701, with a sensitivity of 0.903 and a specificity of 0.459 (Table 3). Similarly, the calibration curve and the Hosmer-Lemeshow test showed good consistency between the predicted and actual outcomes (*p* > 0.05) (Figure 5D). The DCA also confirmed the high clinical utility of the SVM model (Figure 5D).

**Table 3:** Performance indicators of the model.

Group	AUC	95%CI	Sensitivity	Specificity	ACC	PPV	NPV	Brier Score
LR-Training set	0.778	0.669–0.886	0.935	0.514	0.706	0.617	0.905	0.19
LR-Validation set	0.772	0.662–0.882	0.968	0.459	0.691	0.6	0.944	0.193
SVM-Training set	0.729	0.609–0.849	0.968	0.486	0.706	0.612	0.947	0.217
SVM-Validation set	0.701	0.576–0.825	0.903	0.459	0.662	0.583	0.85	0.219

ACC, accuracy; PPV, positive predictive value; NPV, negative predictive value.

The difference analysis of radiomics scores output by the LR model and SVM model significantly differed in terms of the distribution of radiomics scores between the CD74 high and low groups (*p* < 0.05). As depicted in Figures 5E,F, the CD74 high expression group exhibited higher radiomics scores.

The DeLong test was used to compare the AUC values of the LR model and SVM model before and after cross-validation. The results indicated that the *p* value was 0.79 before cross-validation and 0.39 after cross-validation. The AUC values of the LR model and SVM model before and after cross-validation were not significantly different, suggesting that each model has good prediction efficiency.

## Discussion

The classical prognostic indicators of lung cancer are no longer adequate for prognostic stratification and individualized treatment of highly heterogeneous NSCLC (6). Fortunately, radiomics is currently utilized not only for lung cancer diagnosis, assessing the tumor microenvironment, and predicting survival prognosis but also for identification of gene alterations and even prediction of gene expression (37, 38). Based on this premise, we developed a machine learning-based radiomics model that successfully predicted the expression of CD74 in the tumor microenvironment of NSCLC and established the relationship between enhanced CT radiomics features and tumor prognosis. The radiomics features of the machine learning model included large area high gray level emphasis, maximum probability, and zone variance. The feature scores output by the model can effectively distinguish the level of CD74 expression, providing a new indicator for prognosis stratification and individualized precision treatment of lung cancer patients.

Many studies have confirmed the close relationship between the expression of CD74 and the occurrence and development of tumors. For instance, several studies have found a positive correlation between CD74 and MHC class II molecule expression, leading to a higher overall survival rate in certain tumor patients (39–41). Moreover, other studies have indicated that CD74 promotes tumor proliferation and that its expression is negatively correlated with patient survival (10, 42). However, due to significant biological differences among different malignancies, there may not be a uniform answer regarding the role of CD74 in various tumors. Our analysis of both the NSCLC dataset and LUAD dataset revealed that high expression of CD74 is an independent prognostic factor for improved survival. Additionally, GSVA analysis demonstrated the activation of multiple tumor pathways in the CD74 low expression group. These findings suggest that CD74 can serve as a prognostic biomarker in NSCLC.

CD74 plays an important role in several key processes of the immune response, including antigen processing, endocytic maturation, cell migration and signal transduction (43). One study found that high expression of CD74 enhances the immune function of macrophages and CD8+ T cells in the tumor microenvironment of hepatocellular carcinoma. Additionally, high expression of CD74 is an independent predictor of good prognosis in patients with hepatocellular carcinoma (44). In our study, we observed high expression of antitumor-associated antigen processing and presentation genes in the tumor microenvironment of NSCLC patients with high CD74 expression. We also found that CD74 promotes the infiltration of macrophages, memory B cells, and CD8+ T cells in the tumor microenvironment. Macrophages 1 and CD8 + T cells are the main antitumor immune cells in the tumor microenvironment (45, 46). Furthermore, studies have shown that CD74 can be rapidly internalized on tumor cells, making it a determining factor for conjugated chemotherapy or radioisotope carriers. This presents CD74 as a promising target for antibody–drug conjugates (47). A preclinical study demonstrated that the combination of radioisotopes, doxorubicin, amphibian cytosolic ribonuclease ranpirnase, and milatuzumab significantly improved the survival of human malignant tumor xenograft mice and was well tolerated (13). Several studies have also suggested that CD74 is a therapeutic target for milatuzumab (12) and a therapeutic tool for vaccine therapy of malignancies (48). Based on the findings of these studies, it can be inferred that overexpression of CD74 may serve as a potential therapeutic modality.

CT imaging is an essential examination for the clinical diagnosis of lung cancer; however, it lacks objectivity and quantification. Radiomics is a high-throughput “imaging sequencing” data technology that can obtain many imaging parameters and dynamically detect and quantitatively reflect tumor characteristics in a noninvasive way (49). For instance, one study utilized paired radiomics data and RNA sequencing data to unveil the biological significance of radiomics phenotypes for glioblastoma prognosis (50). Another study used head and neck enhanced CT radiomics features to predict the expression levels of prognosis-related molecules in head and neck squamous cell carcinoma (38). Some scholars have also compared machine learning models, including logistic regression, random forest, naive Bayes, SVM, AdaBoost, and neural network models, based on MRI texture features to predict occult lymph node metastasis in early tongue squamous cell carcinoma and confirmed that machine learning models can be an effective predictive tool (51).

Due to the exploratory stage of this study, there are still some limitations. First, due to the complexity of valid data collection, this study did not perform external validation on an independent image dataset to provide further confirmation of the reliability of the model. Second, our data were obtained from an open public database, and the CT image quality was not consistent. Third, the data revealed a mismatch, particularly in the proportion of squamous carcinoma and adenocarcinoma. Fourth, the number of samples is relatively small, and there is a lack of multicentre prospective radiomics studies to guide clinical practice. Increasing the number of CT images from multiple institutions in the future will improve the stability and generalizability of the model. Additionally, adopting standardized methods that meet the Image Biomarker Standardisation Initiative (IBSI) criteria may allow the radiomics model to become a clinically meaningful tool.

## Conclusion

CD74 expression is identified as an independent prognostic factor that significantly affects the overall survival of patients with NSCLC. The enhanced CT radiomics model demonstrates a favorable level of stability and diagnostic efficiency in predicting CD74 expression. This finding suggests that the radiomics model may have the potential to be utilized as a novel method for individualized precision treatment of NSCLC.

## Data Availability

The original contributions presented in the study are included in the article/Supplementary material, further inquiries can be directed to the corresponding author.

## Footnotes

^1^ https://www.ncbi.nlm.nih.gov/geo/query/acc.cgi?acc=GSE103584

^2^ https://portal.gdc.cancer.gov/

^3^ https://www.gsea-msigdb.org/gsea/msigdb/index.jsp

^4^ https://cibersortx.stanford.edu/

^5^ https://pyradiomics.readthedocs.io/en/latest/
