# Supplementary material for: Prognostic effect of CD74 and development of a radiomic model for predicting CD74 expression in non-small cell lung cancer
Source: Front Med (Lausanne). 2025 May 21;12:1586253. doi: 10.3389/fmed.2025.1586253 (PMC12133499; doi:10.3389/fmed.2025.1586253)
Supplement: Supplementary file 2 [file Data_Sheet_2.docx]

***Supplementary Material***

# 1.Supplementary Tables

**Table S1. NSCLC Cohort inclusion and exclusion criteria**

| **NSCLC Cohort** | | | |
| --- | --- | --- | --- |
| **NSCLC-GEO Radiogenomics Clinical Data** | **Exclude** | **Retention** | |
| Total cases | — | | 211 |
| Survival time less than 30 days was excluded | 10 | | 201 |
| Histology type was excluded as unknown | 4 | | 197 |
| T stage was excluded as unknown | 45 | | 152 |
| Samples lacking RNA-seq were excluded | 30 | | 122 |
| **NSCLC-TCIA Radiogenomics Enhanced CT** |  | |  |
| Total cases | — | | 211 |
| The unqualified image was excluded | 104 | | 107 |
| Samples with no intersection of clinical data  and RNA-seq were excluded | 39 | | 68 |

**Table S2. LUAD Cohort inclusion and exclusion criteria**

| **LUAD Cohort** | | | |
| --- | --- | --- | --- |
| **TCGA-LUAD Clinical Data** | | **Exclude** | **Retention** |
| Total cases | — | | 522 |
| Non-primary and non-first diagnosed samples were excluded | 4 | | 518 |
| Missing follow-up data were excluded | 9 | | 509 |
| Survival time less than 30 days was excluded | 14 | | 495 |
| Missing clinical data were excluded | 154 | | 327 |
| Samples lacking RNA-seq were excluded | 7 | | 320 |

**TableS3. Demographic and clinicopathological characteristics of patients (LUAD)**

| **Variables** | **Total (n = 320)** | **Low (n = 166)** | **High (n = 154)** | **p** |
| --- | --- | --- | --- | --- |
| **Gender, n (%)** |  |  |  | 0.002 |
| Female | 172 (54) | 75 (45) | 97 (63) |  |
| Male | 148 (46) | 91 (55) | 57 (37) |  |
| **Age, n (%)** |  |  |  | 0.069 |
| ≤65 | 153 (48) | 88 (53) | 65 (42) |  |
| ＞65 | 167 (52) | 78 (47) | 89 (58) |  |
| **Smoking_status, n (%)** |  |  |  | 0.021 |
| Nonsmoker | 45 (14) | 18 (11) | 27 (18) |  |
| Current | 83 (26) | 53 (32) | 30 (19) |  |
| Former | 192 (60) | 95 (57) | 97 (63) |  |
| **T_stage, n (%)** |  |  |  | < 0.001 |
| T1 | 108 (34) | 39 (23) | 69 (45) |  |
| T2 | 173 (54) | 105 (63) | 68 (44) |  |
| T3/T4 | 39 (12) | 22 (13) | 17 (11) |  |
| **N_stage, n (%)** |  |  |  | 0.06 |
| N0 | 211 (66) | 101 (61) | 110 (71) |  |
| N1/N2/N3 | 109 (34) | 65 (39) | 44 (29) |  |
| **M_stage, n (%)** |  |  |  | 0.956 |
| M0 | 228 (71) | 119 (72) | 109 (71) |  |
| M1/MX | 92 (29) | 47 (28) | 45 (29) |  |
| **Radiotherapy, n (%)** |  |  |  | 0.279 |
| NO | 288 (90) | 146 (88) | 142 (92) |  |
| YES | 32 (10) | 20 (12) | 12 (8) |  |
| **Chemotherapy, n (%)** |  |  |  | 0.814 |
| NO | 213 (67) | 109 (66) | 104 (68) |  |
| YES | 107 (33) | 57 (34) | 50 (32) |  |
| **Residual_tumor, n (%)** |  |  |  | 0.897 |
| R0 | 306 (96) | 158 (95) | 148 (96) |  |
| R1/R2 | 14 (4) | 8 (5) | 6 (4) |  |

## 2. Supplementary Figures

**Figure S1**


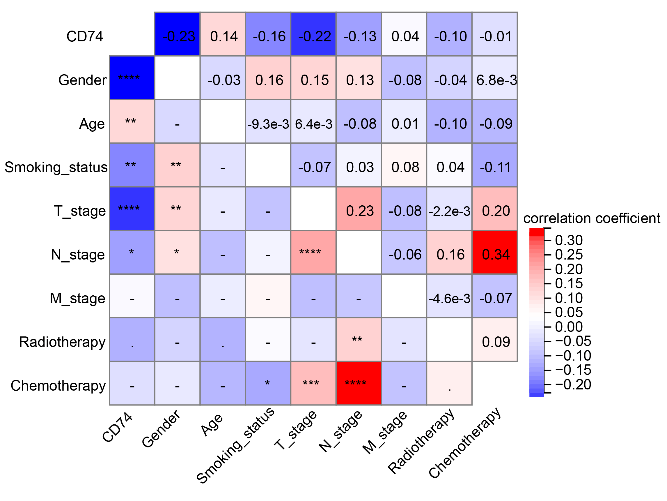


**Fig S1.** Heatmap of correlation between CD74 and clinical features, red represents positive correlation, blue represents negative correlation, and the higher the degree of color, the more significant the correlation. (Significant symbol: -, p≥0.05; *, p< 0.05; **, p<0.01; ***, p<0.001; * * * *, p < 0.0001).

**Figure S2**


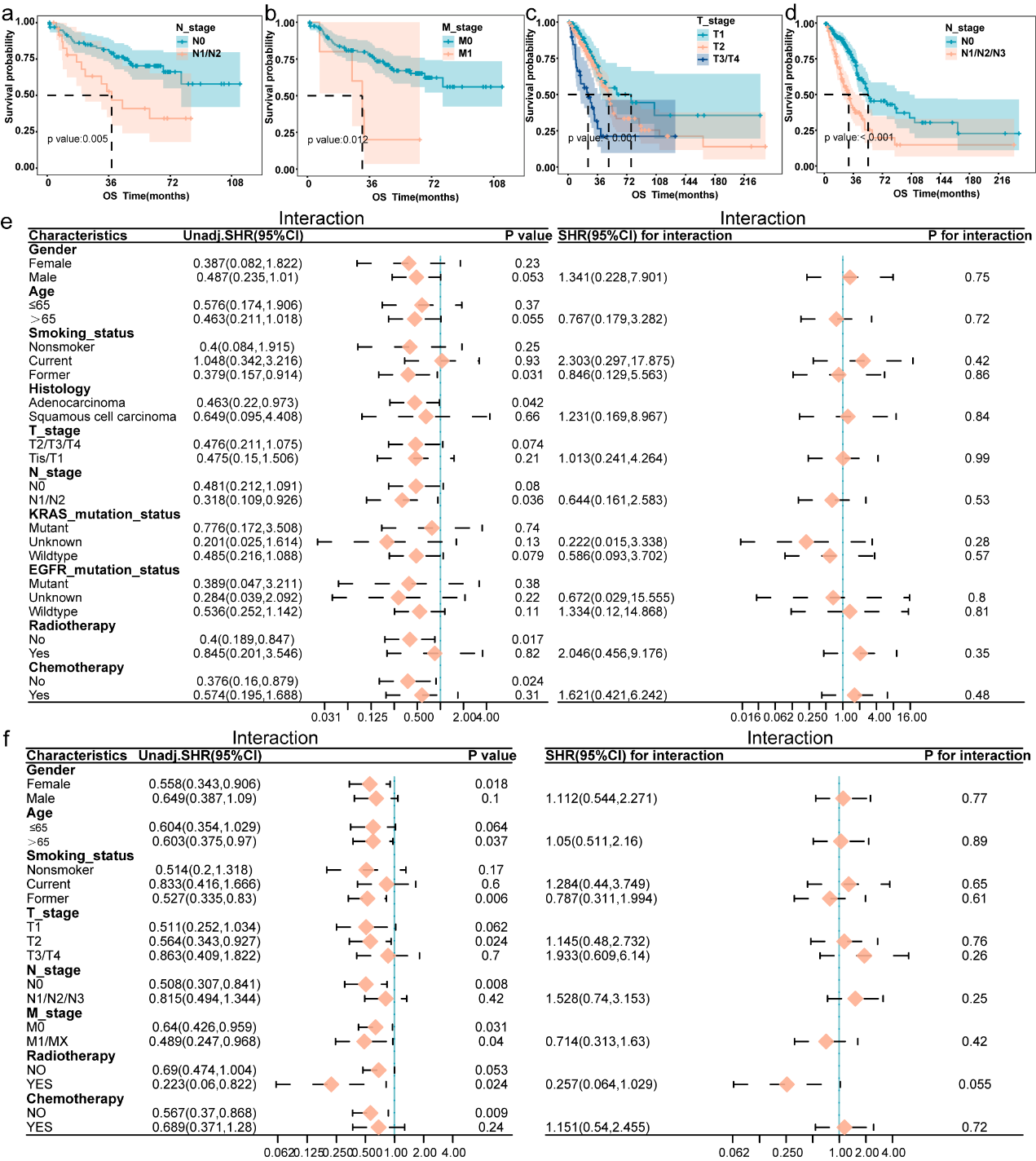


**Fig S2.** Survival Analysis **a.** KM curve of the relationship between N_stage and OS in the NSCLC cohort. **b.** KM curve of the relationship between M_stage and OS in the NSCLC cohort. **c.** KM curve of the relationship between T_stage and OS in the LUAD cohort. **d.** KM curve of the relationship between N_stage and OS in the LUAD cohort. **e.** Forest plots of univariate COX regression analysis and subgroup interaction tests between CD74 groups for each covariate in the NSCLC cohort. **f.** Forest plots of univariate COX regression analysis and subgroup interaction tests between CD74 groups for each covariate in the LUAD cohort.

**Figure S3**


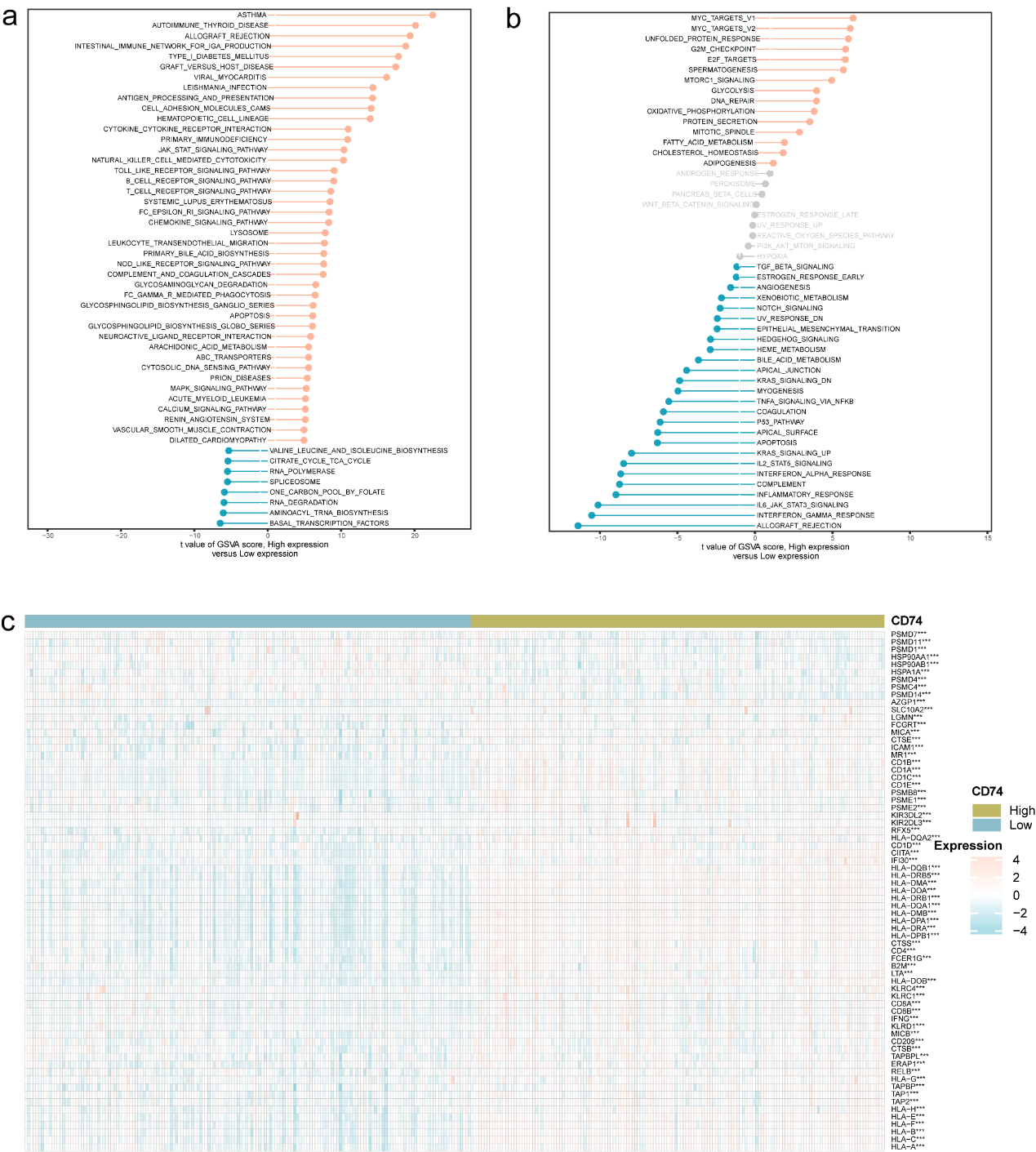


**Fig S3.** Correlation analysis of CD74 tumor pathway and immune response. **a.** GSVA showed differences in KEGG pathway enrichment between CD74 high and low expression groups. **b.** GSVA showed differential enrichment of Hallmarks pathways between CD74 high and low expression groups. **c.** Heat map of differential expression of antigen processing and presentation genes between CD74 high expression group and CD74 low expression group, (significant symbol: *, p< 0.05; **, p<0.01, ***, p<0.001).
